# Diffuse Large B-cell Lymphoma of the Breast Presenting 40 Years After Breast-Conserving Therapy: A Case Report

**DOI:** 10.7759/cureus.107037

**Published:** 2026-04-14

**Authors:** Georgia Xanthopoulou, Christos Barkolias, Kyriaki Theodorolea, Ilektra Spyrou, Nikolaos P Tasis

**Affiliations:** 1 Department of Surgery, Naval and Veterans Hospital of Athens, Athens, GRC

**Keywords:** b-cell lymphoma, breast cancer, breast lymphoma, breast radiation, radiation-induced neoplasm

## Abstract

Breast-conserving surgery combined with radiotherapy has been established as a standard therapeutic option for the management of early-stage breast cancer. Although radiation-associated tumors most frequently include sarcomas, lung cancer, and contralateral breast carcinoma, lymphoid malignancies arising within previously irradiated breast tissue are rarely reported. Breast lymphoma is a rare entity, and its occurrence following prior treatment for breast cancer may pose a diagnostic challenge as it can mimic recurrence of the primary malignancy. We report a case of diffuse large B-cell lymphoma of the breast in an 88-year-old woman presenting approximately 40 years after breast-conserving surgery and radiotherapy for invasive ductal carcinoma. The lesion was initially suspected to represent recurrent breast cancer based on clinical and imaging findings; however, histopathological and immunohistochemical evaluation confirmed lymphoma. Although the tumor arose within a previously irradiated field, a causal relationship with prior radiotherapy could be established and should be interpreted with caution. This case highlights the importance of considering alternative diagnoses in patients presenting with new breast lesions long after initial cancer treatment, particularly in elderly individuals.

## Introduction

Breast lymphoma is a rare entity representing approximately 0.04-0.5% of malignant breast tumors and less than 2% of extranodal non-Hodgkin lymphomas [1,2]. It may occur as primary breast lymphoma (PBL), where the breast is the principal site of disease, or as secondary breast lymphoma (SBL) associated with systemic disease [3]. Diffuse large B-cell lymphoma is the most common histologic subtype [4]. Clinically and radiologically, breast lymphoma frequently presents as a unilateral breast mass and is often indistinguishable from primary breast carcinoma, making histopathological confirmation essential for its challenging diagnosis [5].

Breast-conserving surgery followed by radiotherapy is widely used in the treatment of early-stage breast cancer and has substantially improved long-term outcomes. However, radiation is known to increase the risk of secondary malignancies, particularly after long latency periods. Although breast radiation-associated tumors usually include sarcomas, lung cancer, and contralateral breast carcinoma, lymphoid malignancies arising within previously irradiated breast tissue are rarely reported [6,7]. We report a case of B-cell lymphoma developing in the breast approximately 40 years after breast-conserving surgery and radiotherapy in an 88-year-old female patient, highlighting the importance of considering alternative malignancies in patients presenting with a new breast lesion long after initial cancer treatment.

## Case presentation

**Figure 1:**
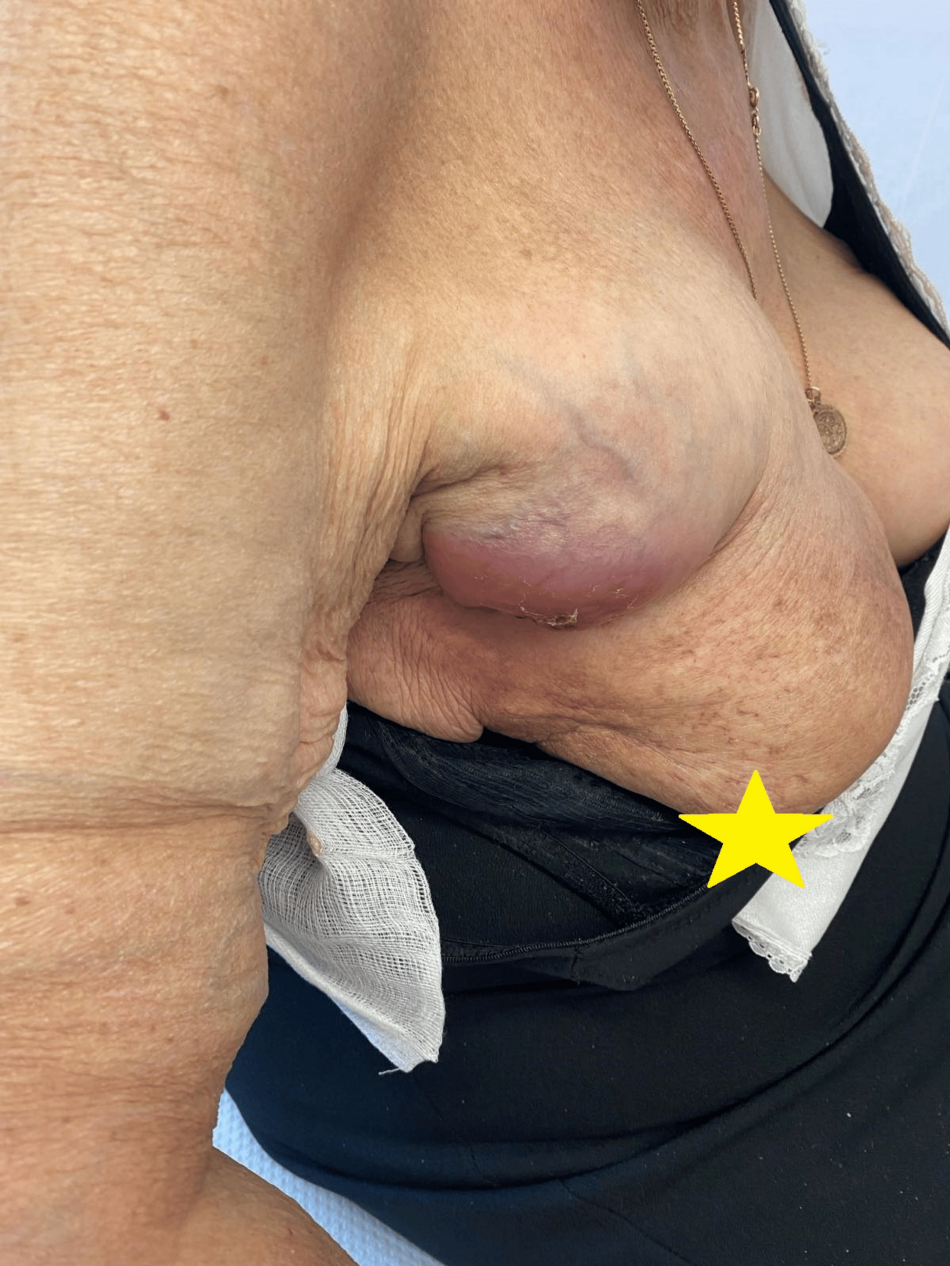
Right breast mass with local erythema

An 88-year-old female patient presented with a two-month history of a progressively enlarging, painful mass in the right breast (Figure 1). Her past oncologic history was notable for invasive ductal carcinoma of the right breast diagnosed at the age of 48 years, which had been treated with breast-conserving surgery followed by adjuvant radiotherapy. Due to the long interval, detailed information regarding the radiotherapy technique and dose was not available. No evidence of recurrence had been documented during the subsequent four decades. 

Her past medical history included arterial hypertension, well-controlled with medication. She reported no history of smoking or alcohol consumption and had no known drug allergies. At presentation, her performance status was assessed as Eastern Cooperative Oncology Group (ECOG) grade 3 [8], as she was capable of only limited self-care and was confined to bed or chair for more than 50% of waking hours, requiring significant assistance from caregivers. On physical examination, a tender palpable mass measuring approximately 7 cm in diameter was identified in the upper outer quadrant of the right breast. The overlying skin demonstrated localized erythema and tenderness, without ulceration. No nipple retraction, discharge, or other nipple abnormalities were observed. Diffuse palpable axillary lymphadenopathy was present on clinical examination.

Bilateral mammography revealed a 7,5 cm mass in the upper outer quadrant of the right breast, corresponding to the clinically palpable lesion. Breast ultrasound confirmed the presence of a solid mass measuring approximately 7 cm in the same location, along with suspicious axillary lymph nodes. Given the patient’s previous history of breast carcinoma, the lesion was initially suspected to represent a locally advanced recurrence of breast cancer. A core needle biopsy of the breast mass was performed.

**Figure 2:**
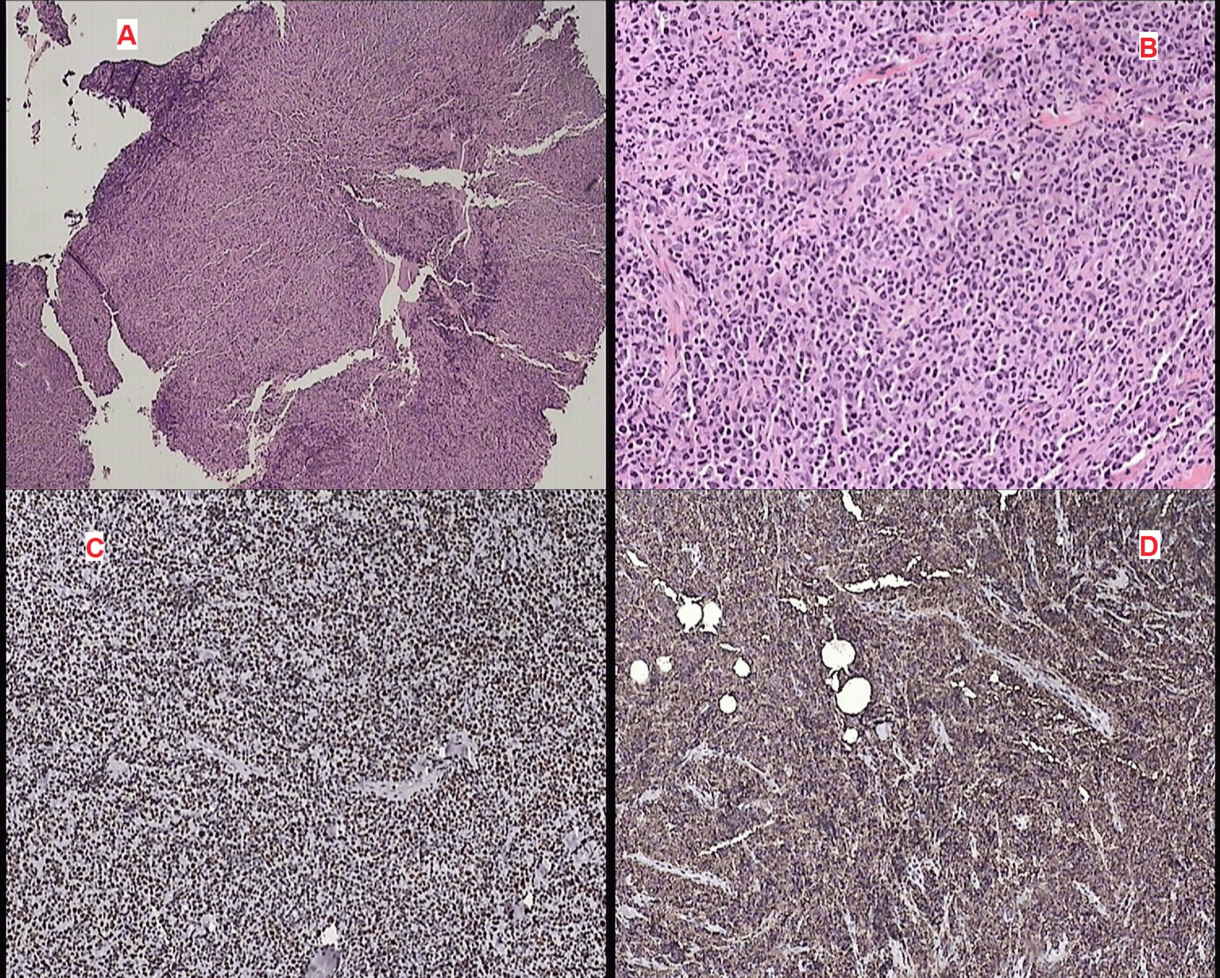
Histopathological and immunohistochemical features of the breast lesion (A) Hematoxylin and eosin (H&E) stain (×4) demonstrating diffuse infiltration of the breast parenchyma by a dense lymphoid population. (B) Hematoxylin and eosin (H&E) stain (×20) showing sheets of large atypical lymphoid cells with pleomorphic nuclei, prominent nucleoli, and increased mitotic activity, consistent with high-grade lymphoma. (C) Ki-67 immunostaining demonstrating a high proliferative index, indicating aggressive tumor behavior. (D) CD20 immunohistochemistry showing strong diffuse membranous positivity, confirming B-cell lineage.

Histopathological examination (Figure 2) demonstrated dense lymphoid infiltration replacing the normal breast parenchyma, composed of large atypical lymphoid cells with features suggestive of non-Hodgkin lymphoma. Immunohistochemical analysis showed strong positivity for CD20 and CD79a, confirming B-cell lineage. Additional staining revealed LCA positivity and vimentin positivity, while CD10 was negative. The tumor cells also expressed BCL2 and BCL6 (>30%), with a high proliferative index (Ki-67 approximately 80%). The neoplastic cells were negative for epithelial and breast carcinoma markers, including pankeratin, estrogen receptor (ER), progesterone receptor (PR), and HER2/cerbB2, as well as chromogranin. The immunophenotypic profile was consistent with diffuse large B-cell lymphoma (DLBCL) involving the breast.

**Figure 3:**
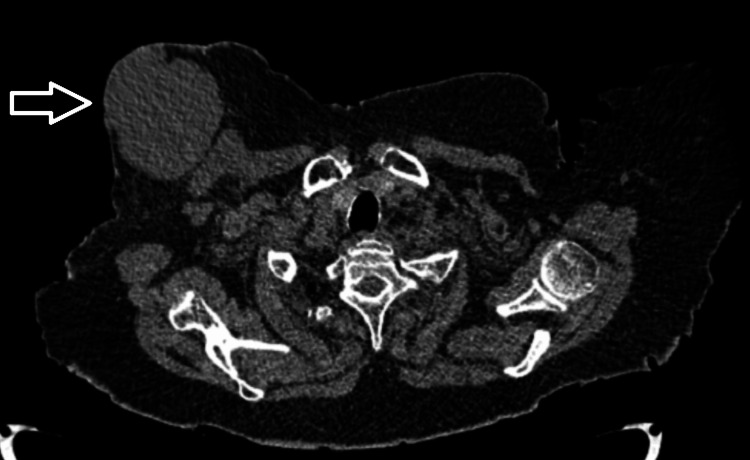
CT scan showing the right breast mass (arrow)

For staging purposes, contrast-enhanced computed tomography of the thorax and abdomen was performed (Figure 3) and demonstrated an 8 cm mass and multiple ipsilateral enlarged lymph nodes, and no evidence of distant metastatic disease. According to the Ann Arbor classification [9], the disease was consistent with stage IIE. Thus, the case fulfills the Wiseman and Liao criteria [10] for primary breast lymphoma, including absence of prior lymphoma, localization to the breast with ipsilateral lymph node involvement, and histopathologic confirmation. The International Prognostic Index (IPI) score was 3, based on advanced age (>60 years), elevated lactate dehydrogenase levels, and impaired performance status (ECOG 3), corresponding to a high-intermediate risk category. Although the lymphoma arose within a previously irradiated field, any causal relationship with prior radiotherapy remains uncertain and should be interpreted with caution. The long latency period observed in this case may be consistent with radiation-associated malignancy [6]; however, this finding could also be coincidental, particularly given the patient’s advanced age [11].

Following the histopathological diagnosis, the patient was referred to the hematology-oncology service for evaluation of systemic treatment. A pre-treatment cardiologic assessment revealed previously undiagnosed heart failure with a reduced ejection fraction (<40%), necessitating further evaluation. Given the patient’s advanced age, impaired cardiac function, and overall performance status, she was deemed unfit for systemic chemotherapy by the multidisciplinary team. Consequently, no oncologic treatment was initiated, and management was focused on supportive and palliative care. The disease subsequently progressed clinically, and the patient deteriorated, ultimately dying four months after the initial diagnosis due to disease progression.

## Discussion

Breast lymphoma is a rare malignancy that typically presents with nonspecific clinical and imaging findings, most commonly as a palpable breast mass that may occasionally be associated with pain, breast enlargement, or localized erythema [2,12]. Radiologic evaluation with mammography or ultrasonography usually reveals an ill-margined, hypoechoic mass without specific characteristics [1,5]. Consequently, imaging alone cannot reliably distinguish lymphoma from primary breast carcinoma or other primary of metastatic breast malignancies, and histopathologic evaluation with immunohistochemical analysis remains the gold standard for the diagnosis [2,12].

The diagnostic challenge becomes particularly significant in patients with a previous history of breast carcinoma, where newly detected breast lesions are often presumed to represent locoregional recurrence. Several reports have shown that breast lymphoma may closely mimic recurrent carcinoma both clinically and radiologically, potentially delaying the correct diagnosis [3,12]. In the present case, the patient presented with a rapidly enlarging breast mass four decades after treatment for invasive ductal carcinoma, and the initial clinical suspicion was consistent with locally recurrent breast cancer. However, core needle biopsy with immunohistochemical analysis demonstrated a lymphoid neoplasm expressing B-cell markers such as CD20 and CD79a, with high proliferative activity and absence of epithelial markers, findings characteristic of diffuse large B-cell lymphoma [11].

PBL is an uncommon extranodal lymphoma, and its pathogenesis remains incompletely understood. Several mechanisms have been proposed, including malignant transformation of lymphoid cells within intramammary lymph nodes or lymphatic tissue, as well as chronic immune stimulation [5,12]. Importantly, advanced age is a well-established risk factor for diffuse large B-cell lymphoma and likely represents the most significant contributing factor in this case [11]. The occurrence of lymphoma in elderly patients is generally considered multifactorial, reflecting cumulative genetic alterations, immune senescence, and environmental influences.

Secondary malignancies following radiotherapy are well documented and may occur many years after exposure due to the mutagenic effects of ionizing radiation on normal tissues. Epidemiologic studies have demonstrated that the risk of second cancers may persist for decades after treatment [6,13]. However, the malignancies most commonly associated with prior breast irradiation include angiosarcoma, lung cancer, esophageal cancer, and contralateral breast carcinoma [6,7]. In contrast, hematologic malignancies, and particularly lymphomas arising within previously irradiated breast tissue, are rarely reported.

Although the localization of lymphoma within a previously irradiated field in this case is noteworthy, any causal relationship with prior radiotherapy remains uncertain and cannot be established. The observed association may be coincidental, particularly given the patient’s advanced age and the known epidemiology of diffuse large B-cell lymphoma [12,14]. Large epidemiologic studies have demonstrated that the relative risks associated with radiotherapy for second malignancies tend to decrease with increasing age at initial cancer diagnosis, while increasing with longer follow-up intervals [6,7]. This suggests a complex and multifactorial relationship between radiation exposure and secondary cancer development, in which age remains a significant modifying factor without excluding the role of previous regional radiotherapy. Previous reports describing lymphoma arising in treated breast tissue are limited and consist mainly of isolated case reports or small series [13], with latency periods typically ranging from several years to over a decade. The approximately 40-year interval observed in the present case is exceptionally long and further supports the need for cautious interpretation.

From a clinical perspective, this case highlights the importance of maintaining a broad differential diagnosis when evaluating new breast lesions in long-term breast cancer survivors. Imaging findings alone may be insufficient to differentiate lymphoma from recurrent carcinoma, and therefore tissue diagnosis remains indispensable, particularly because the management of breast lymphoma differs fundamentally from that of breast carcinoma and relies primarily on systemic chemotherapy rather than surgical treatment [2,11].

## Conclusions

Diffuse large B-cell lymphoma of the breast is a rare malignancy that can closely mimic recurrent breast carcinoma in patients with a prior history of breast cancer. Because clinical and imaging findings are often nonspecific, histopathologic evaluation with immunohistochemistry remains essential to establish the correct diagnosis and guide appropriate management.

The present case describes the occurrence of breast lymphoma four decades after breast-conserving therapy, representing an unusually long latency period. Although the tumor arose within a previously irradiated field, a causal relationship with prior radiotherapy cannot be established and should be interpreted with caution. The development of lymphoma in this patient is likely multifactorial, with advanced age representing a more established contributing factor. This case underscores the importance of maintaining a broad differential diagnosis when evaluating new breast lesions in long-term breast cancer survivors.
